# Fungi as a model for expanding the understanding of dormancy across microbial life

**DOI:** 10.1128/msystems.01014-26

**Published:** 2026-09-15

**Authors:** Danae Bregnard, Saskia Bindschedler, Simona Regenspurg, Pilar Junier

**Affiliations:** 1 Laboratory of Microbiology, University of Neuchâtel 27214, Neuchâtel, Switzerland; 2 Geoenergy, GFZ Helmholtz Centre for Geosciences 28337, Potsdam, Germany; CNRS Delegation Alpes, Villeurbanne, Rhône-Alpes, France

**Keywords:** fungi, bacteria, dormancy, spores, conidia, ascospores, scletoria

## Abstract

Dormancy is a widespread strategy that enables microorganisms to survive unfavorable environmental conditions. While bacterial dormancy has been extensively studied, a comprehensive overview of fungal dormancy is missing, despite its ecological, medical, and industrial relevance. In this minireview, we summarize the diversity of structures in fungi that could play a role in dormancy and highlight their morphophysiological complexity, emphasizing their distinct developmental pathways and resistance mechanisms. Whenever possible, these structures are presented alongside a brief description of dormant bacterial cells to illustrate potential parallels between both groups and to highlight commonalities. We also explore the challenges of detecting and identifying fungal dormant structures in environmental samples, with a focus on our own research in deep geothermal environments. Despite recent advances, significant methodological gaps remain, particularly in the detection of structures like sclerotia. Recognizing the diversity of dormant structures and integrating multiple microbial models is essential to develop a unified framework for dormancy research. Such integration will enhance our understanding of microbial survival strategies and their roles in ecosystem resilience, biogeochemical cycling, and applied contexts ranging from food safety to human health. By identifying both shared and unique features of dormancy across microbial domains, the ecological significance of this fundamental strategy can be better explained and explored.

## INTRODUCTION

A common theme for life across complex scales and diverse habitats and ecologies is the “fight” for survival. For some organisms, this fight will involve developing strategies that allow them to cope with conditions that hinder or limit growth, such as nutrient scarcity, extreme temperatures, desiccation, or the presence of toxic compounds. These strategies can also include escaping predators or even migrating and colonizing more suitable habitats. Becoming dormant is one strategy that allows organisms to respond to all of the above challenges (1). The definition of dormancy has varied across scientific subdisciplines and has evolved over time. In a recent opinion article on the topic, McDonald et al. (2) define dormancy as “a temporary, adaptive state of reduced metabolic activity within an extended period of arrested growth that enables a microbe to maintain viability under unfavorable environmental conditions.” This definition underlines the commitment to a specialized metabolic state that will act as a response to “wait it out,” until conditions are once more favorable for growth (3). However, given that diverse ecosystems present unique sets of challenges for survival, this behavior should be tailored to respond to specific environmental cues and is likely expressed in a wide variety of survival traits. Accordingly, dormancy can be expected to manifest differently across taxonomically and functionally distinct microbial groups.

The term “microbe” encompasses varied taxonomic groups, from viruses to prokaryotes and microeukaryotes. While dormancy has been extensively reviewed in bacteria, this has not been the case in eukaryotic microbes. Given that the fungal kingdom has recently gained increased interest due to its now appreciated roles in fields as varied as soil fertility or human health (4), this review proposes to comprehensively explore the diversity of dormancy strategies in fungi. This microbial eukaryote taxon holds a large diversity of potential dormant structures, combined with complex life cycles, thereby offering a unique opportunity to examine the definition of dormancy as a unified concept in microbiology. Research on fungal dormancy has been strongly influenced by the traditional use of the term in plant science. In this case, dormancy is applied for viable seeds that are incapable of germinating during a certain period of time under favorable external conditions (5). Based on this, the problems of defining dormancy of fungal spores were already extensively discussed in an article by Cochrane in the 70s (6). The operational definition of fungal dormancy as a period of rest or after-ripening before spores can germinate does not consider many of the current elements that define a dormant cell and should be accordingly adapted to the current knowledge in the field. More recently, Wyatt et al. (7) proposed a review on fungal spores where they differentiate two types of dormancy responses: exogenous dormancy, acting as a response to environmental stressors, and constitutive dormancy, which corresponds to a metabolic control of dormancy and thus matches the initial definition proposed by Cochrane. In the case of constitutive dormancy (sometimes referred to as endogenous dormancy), experiments have shown that germination is triggered by a process called “cytoplasmic regulation” that does not rely on a nuclear signal. The information required to go out of the dormant state is transferred even before the dormant cell is fully formed. Breaking dormancy is accordingly not only linked to changes in environmental conditions but requires overcoming a system of cellular barriers and metabolic blocks. In contrast, exogenous dormancy can be broken when the effect of an unfavorable factor (e.g., water limitation) ceases (8).

The principles of dormancy can be applied to any component of a system (here a cell) provided that criteria such as the cycling and transition between different activity states are fulfilled, along with an increased level of protection from decay while in a state with lesser activity (9). Some of those criteria are intuitively met by fungal spores, for which the elements of state transition are easily observable. However, expanding the concept of dormancy to encompass any rest period or reversible interruption of an organism’s phenotypic development, together with the associated reduction in metabolic activity, substantially broadens the range of fungal structures that may be classified as dormant. These structures extend well beyond spores and may provide important insights for advancing the study of dormancy across other microbial groups.

### Diversity of dormant structures in fungi

Fungi are a clade of eukaryotes that present both unicellular and multicellular forms. They are mostly microscopic organisms, but they also occupy macroscopic scales owing to their 3D filamentous lifestyle. Fungi possess remarkably diverse physiologies, metabolisms, and ecological functions that are mirrored by complex life cycles along with varied adaptive structures to cope with this diversity (10). However, this group is often comparatively less represented than bacteria in general discussions about dormancy and its role in survival. This is despite the ecological and clinical significance of fungal dormant cellular states and the significant body of information concerning dormancy in fungi.

The ability of an organism to enter a reversible state of reduced metabolic activity, which is the core premise of dormancy, requires cells to be able to transition between different metabolic states by a process that results in the formation of a dormant structure (i.e., initiation), offering protection from decay, and another one that allows the organism to exit dormancy (i.e., resuscitation). The confluence of morpho-physiological traits required to fulfill these requirements can result in the production of highly specialized dormant cells (11). Several specialized cells and tissues are produced as part of the life cycle of most fungal species. The function of those cells is not necessarily survival but also reproduction and dispersal. However, the latter usually requires some level of constitutive dormancy until the specialized cell encounters adequate conditions to germinate and resume another vegetative life cycle. Hence, it is important first to describe different potential dormant structures and to consider if they fulfill the definition of dormancy.

#### Fungal spores and shared traits with bacterial spores

Some of the best-understood models of dormant cells are those associated with the specific commitment for differentiation upon cell division that results in a specialized cell called “spore.” Sporulation is a strategy widely utilized by diverse organisms to cope with changes in their individual environmental niches and to survive in time and/or space until they encounter conditions acceptable for vegetative growth. Sporulation occurs across the tree of life in organisms ranging from bacteria, protozoa, plants, and fungi; and while most of what is known about spores and dormancy is from bacterial systems, most of the spore-forming organisms on Earth are fungi (12).

Fungal spores are the most common type of cells when considering fungal dormancy. However, not all fungal spores are involved in dormancy and thus, concerning the general premises of dormancy, it is important to establish if a given spore type corresponds to a transition between active and inactive metabolic states and if they provide increased protection against decay. As indicated above, different definitions of dormancy have been proposed based on the trigger of dormancy (i.e., exogenous versus constitutive) (7).

Many fungal species form different types of spores within their life cycle, and historically, the fungal kingdom has been divided based on morphological traits associated with sexual reproductive structures, including spores and structures linked to spore production (13). In general, fungal sexual spores are considered a form of constitutive or endogenous dormancy, while asexual spores are a form of exogenous dormancy (8). Sexual spores are generally regarded as structures adapted for long-term survival under adverse environmental conditions, whereas asexual spores are primarily specialized for efficient dispersal. However, this distinction is not absolute in all fungal groups, and the resistance to adverse conditions depends eventually on spore ultrastructure and the characteristics of the environment in and from which fungal spores disperse (7).

Sexual spores are the result of the genetic exchange of two contributing individuals that leads to the generation of novel genetic variants (14, 15). In Ascomycota, ascospore morphogenesis is a complex developmental process where sexual spores are formed within a sac-like cell called an ascus. It begins with nuclear fusion (karyogamy) followed by meiosis to produce haploid nuclei, which then undergo mitosis to yield up to eight nuclei that are compartmentalized by double delimiting membranes, or prospore membranes (16). In Basidiomycota, basidiospore morphogenesis involves the development of haploid spores from a binucleate (dikaryotic) mycelium. This happens via karyogamy from a dikaryotic cell which eventually becomes a basidium, followed by meiosis to produce four haploid nuclei. Each nucleus then migrates into a bud that develops into a sterigma carrying a sexual exospore called the basidiospore (17). In contrast, asexual spore or conidia development in Ascomycota and Basidiomycota fungi (also known as Dikarya) is the result of mitotic cell division, eventually producing a clonal cell population (18).

**Fig 1:**
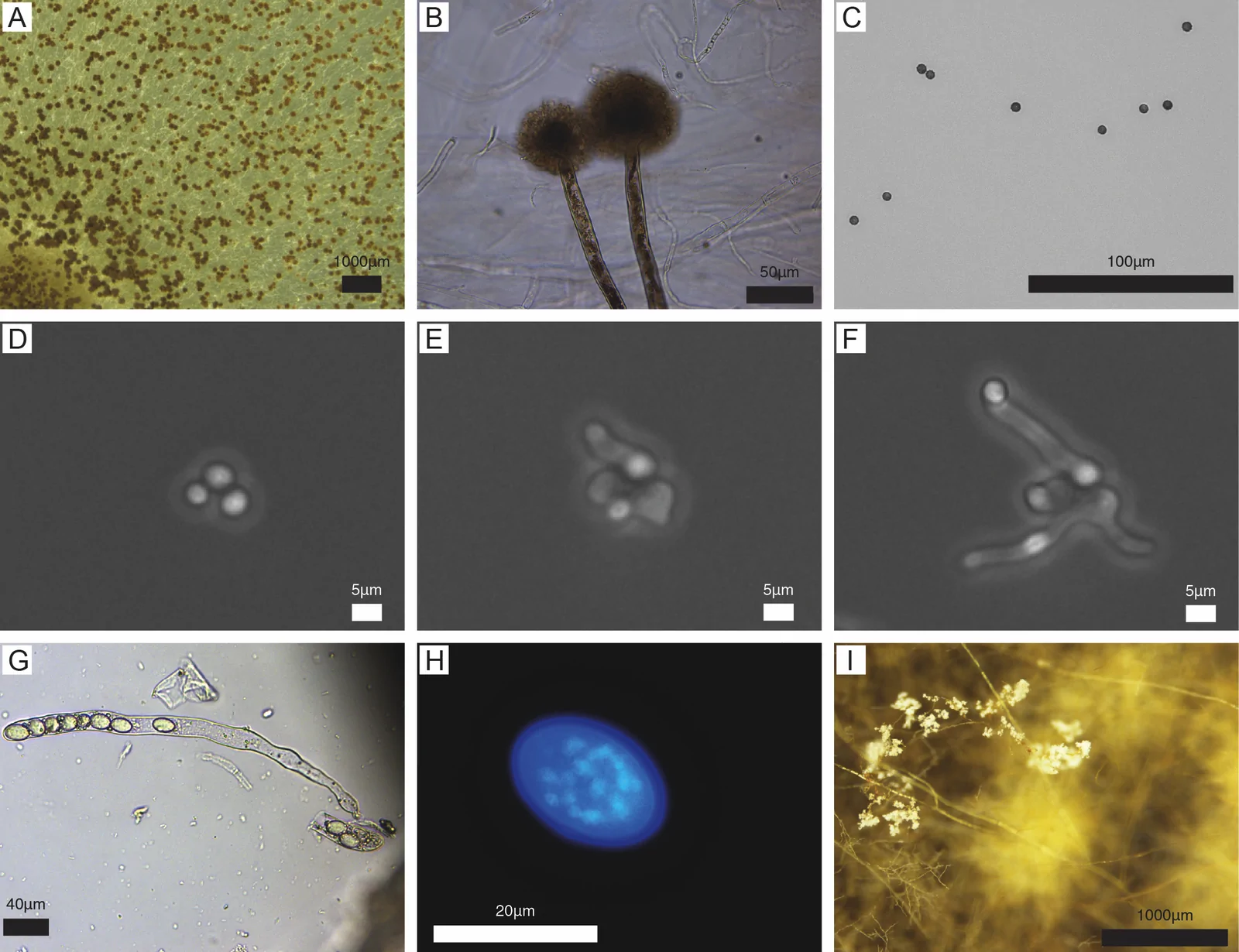
Images illustrating examples of fungal spores. (**A and B)** Conidiophores produced during the asexual phase in the life cycle of *Aspergillus niger.* (**C)** Conidia from the same species. Images (**E and F)** illustrate the process of conidia germination showing swelling (**D**) and hypha emergence (**E and F**). Sexual reproduction in the ascomycete *Morchella* spp. results in the formation of a carpophore in which asci (**G**) contain ascospores (sexual spores). Ascospores can contain multiple nuclei (**H**). Conidia (asexual spores; **I**) contribute to asexual dispersion for the same group. Images provided by Fabio Palmieri (**A-C**), Aureline Bouchard and Apiha Shanmuganathan (**D-F**), and Melissa Cravero (**G-I**).

The life cycles of many fungal species can be used to illustrate the complex interplay between life history, the production of different types of spores, and their role in dormancy. The process has been intensively studied in model fungi such as *Aspergillus nidulans* and *Neurospora crassa*. For instance, during the asexual phase, vegetative hyphae differentiate into conidiophores (Fig. 1A and B), which are usually produced slightly behind the colony border where nutrient acquisition is taking place. These complex multicellular structures produce chains of mitotically derived conidia (19). The formation of a conidiophore involves a change in the growth polarity of a hyphal compartment, as well as in the capacity of specialized conidiogenic cells called phialides to produce uninucleated conidia (Fig. 1C and D) by repeated mitotic divisions and periodic budding cycles (20). These conidia are highly resistant to desiccation and environmental stress, remaining dormant until favorable conditions trigger germination and colony establishment. Conidia may also be formed during the parasexual phase, producing aneuploidic spores, which may be more adapted to local environmental conditions. In addition, conidia that have reverted to haploidy via aneuploidization have been shown to have an enhanced success of adaptation to new environments (21). Conidia production is comparable to the formation of exospores in Actinobacteria, in which synchronous cell division is initiated to produce many identical spores in non-branching aerial hyphae (22). During the sexual phase, under specific environmental cues such as nutrient limitation, *A. nidulans* undergoes sexual development, forming cleistothecia, which are enclosed fruiting bodies that house meiotically derived ascospores. These ascospores contribute to genetic recombination and long-term survival, often being more stress-resistant than conidia (23).

In other models such as the yeast *Saccharomyces cerevisiae*, sexual spore formation is initiated by environmental cues such as nitrogen starvation in the presence of a non-fermentable carbon source. In this model, the sporulation development program couples meiosis to spore biogenesis within a single mother cell that results from the mating of sexually compatible haploid cells. In yeasts, there is no real asexual spore formation since yeast growth is the clonal propagation of a single cell. However, yeast cells may turn into a dormant state that is called quiescence (24).

Among mushroom-forming fungi such as *Coprinopsis cinerea*, sexual spores are produced upon the encounter of sexually compatible mycelium and are released through the gills of fruiting bodies (12). Another remarkable example that highlights the interplay between spore structures, dormancy, and the life cycle is the genus *Morchella* (morels). In *Morchella*, both sexual multinucleated ascospores (Fig. 1E and F) and asexual conidia (Fig. 1G) are supposed to play a significant role in the life cycle and eventually in fruiting body formation. However, many questions remain about the contribution of each of these structures in nature and the importance of dormancy in completing the different steps of the life cycle of morels. In particular, because it is challenging to replicate the entire life cycle under controlled conditions, explaining the complexity of cultivating these highly prized fungi (25).

In fungi outside of the Dikarya, asexual spores are usually considered endospores, meaning the spores are contained within a compartment called the sporangium. However, there are major differences in terms of morphogenesis between the different taxonomic groups. In filamentous aseptate fungi of the Mucoromycota phylum, thousands of asexual spores are usually formed and contained in a sporangium. There, a major exception exists in the Glomeromycotina. This fungal subphylum encompasses arbuscular mycorrhizal fungi (AMF), the most globally widespread type of mycorrhizal fungi. Glomeromycotina form very large, single-celled fungal spores (up to 1 mm in diameter; [26]). These spores originate from a cell compartment, which may be considered a sporangium, whose nuclei never experienced cytoplasmic cleavage, ending up in a multinucleate asexual exospore (27). Dormancy in AMF spores may be considered a mix of endogenous (constitutive) and exogenous triggers (28). Another very important type of spore found in Mucoromycota are zygospores, which are sexual spore. For instance, in *Rhizopus* spp., the morphogenesis of zygospores involves the development of specialized hyphal tips called gametangia, which fuse to form a single zygosporangium containing a thick-walled zygospore. Zygospores undergo a period of dormancy before germinating and undergoing meiosis to produce haploid spores for dispersal (29).

Asexual and sexual fungal spores undoubtedly correspond to a transitional and reversible state that contains the information for the phenotypic development of a growing individual. However, whether they have a reduced metabolic state and if they provide increased protection against decay can be discussed. Regarding the reduced metabolic state, dormant cells are not actively growing; however, even if it is at a lower rate, they might still be metabolically and transcriptionally active. Diverse studies have shown that asexual spores contain a large amount of highly stable transcripts, but the purpose of those is still controversial (30, 31). Interestingly, the mRNA found in conidia still attached to conidiophores of different *Aspergillus* species has been shown to vary in composition and to respond to environmental cues. These differences in composition are expected to lead to conidia that are better suited to their local environment when they exit dormancy. This process allows genetically identical conidia to differ phenotypically and thus optimize their survival after dormancy (32).

Concerning protection against decay, depending on the species, both sexual and asexual spores are known to be at least as resistant and often more resistant to abiotic and biotic stress factors than their corresponding vegetative states (33). For instance, microconidia (asexual spores) of *Fusarium oxysporum* have the same level of resistance to antifungals as vegetative cells (33), while ascospores (sexual spores) of yeasts such as *S. cerevisiae* are significantly more resistant to high temperatures than their vegetative counterparts, exhibiting up to 350-fold greater heat tolerance (34). Moreover, sexual ascospores of some fungal species involved in food spoilage are considered candidates for the most stress-resistant eukaryotic structures. This type of ascospores can survive temperatures used for pasteurization (i.e., 85°C) (35). As indicated earlier, ascospores are thought to be more resistant to various abiotic stresses than conidia (7). Moreover, owing to the importance for plant growth and their potential as bioinoculants, spore characteristics such as morphological traits and resistance have been well characterized in AMF (36). Along with a complex and multi-layered cell wall, the multinucleate nature of AMF spores has been linked to their longevity (37). Taken together, the fragmentary evidence from different models suggests that most fungal spores can reasonably be considered canonical dormant structures, although the limited characterization across many species still prevents broad generalization.

Finally, it is worth highlighting a different type of fungal spore that is not considered dormant. Contrary to the dormant spores described above, zoospores, produced by Chytrids, are specialized cells for active dispersal to find suitable environments for growth (including a suitable host). Chytrid fungi are some of the earliest diverging fungal lineages that retained features of the ancestor of animals and fungi, one of which is a flagellated cell, the zoospore. Chytrids and other zoosporic fungi produce zoospores during both sexual and asexual reproduction. Zoospores are motile cells that lack a cell wall and swim with a motile cilium. Once zoospores turn into a vegetative lifestyle, they lose their flagellum and differentiate a cell wall, a process which has been termed encystment. The use of this terminology could confuse the reader into thinking that during encystment, zoospores turn into a dormant state. However, it is exactly the opposite: the encysted cell is the active form that acquires nutrients from the substrate and/or a host (38). As a result, even though zoospores participate in survival by allowing the colonization of a new niche, they are not dormant cells given their metabolic activity. In addition, vegetative cells provide better protection from decay and are more resistant to environmental stressors (39). Nevertheless, in some Chytrid species, resting sporangia have been described. They represent a dormant phase generally formed because of environmental stress and typically harbor a thick, often multi-layered, and pigmented cell wall (40). However, these structures have not been described in all chytrids and as a result, their overall contribution to dormancy in Chytridimyocota remains overlooked. This is especially interesting given that Chytridiomycota are observed in environments where at least one abiotic stress prevails during part of the year (e.g., extremely dry or extremely wet environments), and accordingly, true zoosporic fungi must deploy strategies to adapt to extreme conditions (41).

#### Other types of fungal dormant structures

Other types of dormant cells result from the modification of the outer layers of a vegetative cell without requiring prior division. This is typically the case of both cysts (42) and akinetes (43) in bacteria, but also of chlamydospores, which are fungal resistant cells that result from a process where a cell differentiates into a thick-walled, resistant structure for survival under unfavorable conditions. The process involves cytoplasmic condensation within a specialized cell, formation of a thick, pigmented or hyaline wall, and accumulation of lipid reserves and RNA. This specialized structure, often larger and with a condensed cytoplasm compared to other hyphal cells, can emerge either at the end or in the middle of a hypha. It can also form within fungal tissues of Agaricomycetes, such as in the case of *Auriscalpium vulgare*, where chlamydospores may be observed in the hyphal mesh that makes the flesh of the cap (44). In the model organism *Candida albicans*, chlamydospores resemble a stable, resting cell and are distinct from the proliferative forms characterized by the yeast or hyphal states, but there is currently no strong evidence that supports their long-term viability (45).

A study performed in the 70s comparing the survival potential of chlamydospores with asexual and sexual spores of diverse ectomycorrhizal fungi revealed that the former provided little survival advantage (46). This study was performed to evaluate the development of pine inoculants and might not be representative of other environmental conditions for which chlamydospores would provide protection from decay. Another example of a fungus in which chlamydospores are used as a tool for the dispersal of a beneficial fungus is the nematophagous *Duddingtonia flagrans*. Chlamydospores of this species serve as feed additives for livestock, survive the passage through the digestive tract, and are excreted with the feces, where they can control the populations of parasitic nematodes (47). The effectiveness of chlamydospores as a vector for dispersal and survival in this example is undeniable. However, a study comparing the level of germination and effectiveness of chlamydospores produced under variable conditions indicates that these traits can be highly variable (48). There is also a negative aspect associated with the production of chlamydospores: the germination of dormant chlamydospores of plant pathogens is known to play a major role in disease severity and incidence. In a recent study on the rice pathogen *Ustilaginoidea virens*, breaking of dormancy and germination of chlamydospores was stimulated by the production of exopolysaccharides in the rhizosphere of the host plant (49). These examples provide an incentive for a better understanding of the mechanisms of formation and reactivation of chlamydospores.

The fungal mycelium can also differentiate into dense multicellular aggregations of resistant fungal tissue called sclerotia that can remain latent in the environment for years. Sclerotia have evolved several times in the fungal kingdom and are among the most morphologically diversified resistant structures. Sclerotia are defined as resting structures made of compact masses of hardened mycelium ranging from sub-millimetric structures (e.g., in *Verticillium* spp. with diameters up to 500 µm [50]) to pluri-centimetric ones (e.g., in *Wolfiporia extensa* with a diameter of up to 30 cm [51, 52]). Three types of sclerotia may be recognized: microsclerotia, pseudosclerotia, and true sclerotia. Microsclerotia are usually smaller than 1 mm and consist of dense aggregations of thick cell-walled and melanized hyphae with poor cell differentiation (53). These structures are encountered in several plant pathogens such as *Sclerotinia* spp. and *Verticillium* spp. and contribute to the difficulty in eradicating these pathogens owing to their resistance to desiccation and UV radiation, and extended survival (up to 6–12 years) (54, 55). Pseudosclerotia is a poorly defined term in mycology that usually refers to a fungal survival structure made up of melanized and condensed mycelia wrapped with plant tissues, plant litter, and/or soil. Several species have been mentioned to be able to form pseudosclerotial structures, ranging from plant pathogens where the pseudosclerotia are formed around and within the fruit tissue (e.g., *Monilinia* spp. [56]) to saprotrophs where the pseudosclerotia engulf dead organic material (e.g., *Stropharia* spp. [57]). Finally, true sclerotia are made of agglomerated hyphae differentiating an outer cortex and an inner medulla although differentiation may be poorly defined in some species. Cortex hyphae are thick-walled and melanized, while medulla hyphae are usually more loose and contain nutritive resources. True sclerotia are usually a few mm to cm in size, but they can reach up to 30 cm in diameter in the wood decay fungus *Wolfiporia cocos* (58).

**Fig 2:**
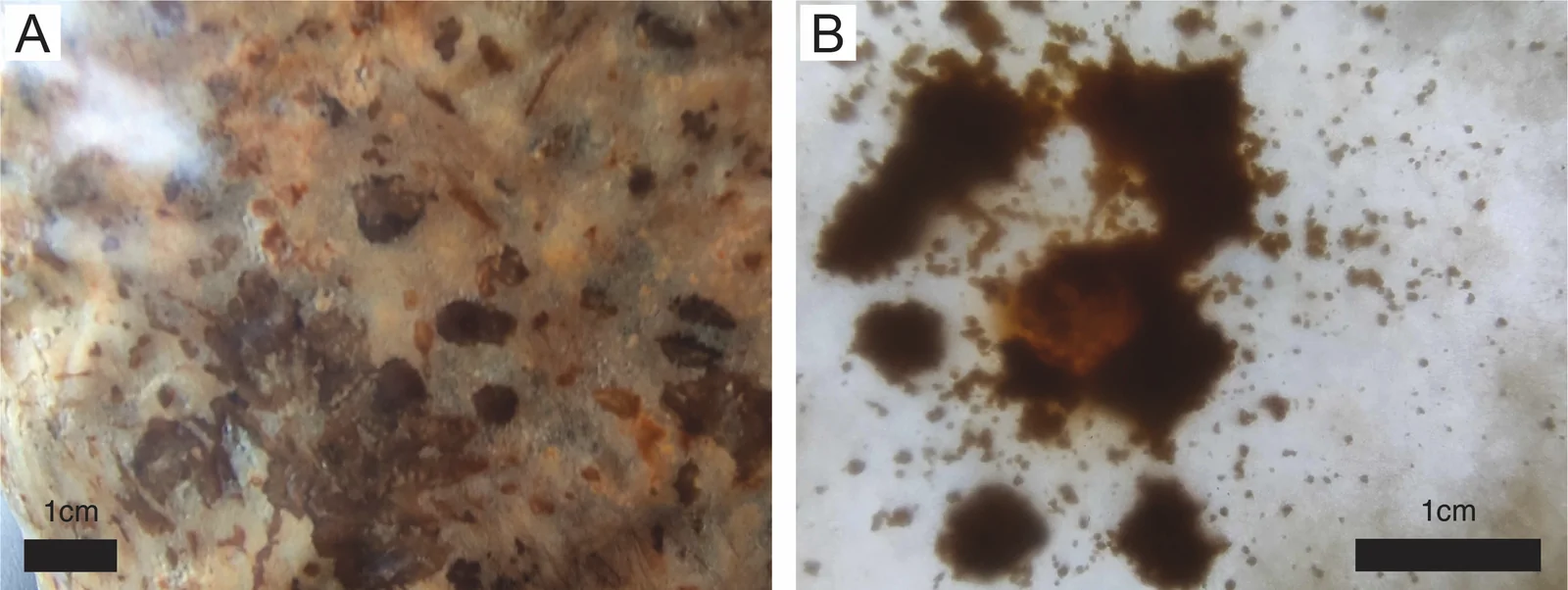
Images of sclerotia in *Morchella* spp. Sclerotia are considered dormant tissues that are supposed to be important in overwinter survival and and carpophore formation. (**A**) Sclerotia in spawn prepared for soil inoculation. (**B**) Sclerotia produced on a malt agar culture. Images provided by Alix Jornot.

In terms of longevity, sclerotia viability is highly variable, depending on the species and environmental parameters. Data available for the plant pathogen *Sclerotinia sclerotiorum* showed a decrease in viability in sclerotia depending on whether they were exposed at the soil surface or not, with viability in the range of <12 months (59). Another study showed a longevity of up to 20 years for sclerotia of another species of *Sclerotinia*, *S. cepivorum* (60). *Aspergillus niger* is another example in which sclerotia function as dormant survival structures. Although less common in laboratory strains of this species, sclerotia are observed in natural isolates and are thought to represent an ancestral trait linked to both survival and sexual reproduction, as they can give rise to cleistothecia under appropriate conditions (61). Finally, sclerotia appear to be an essential structure of the highly prized morel fungi (Fig. 2), as the induction of fruiting bodies from sclerotia is usually considered a prerequisite for the effective cultivation of this fungus under controlled conditions in indoor cultivation systems (62, 63). Importantly, sclerotia allow the fungus to overwinter so that fruiting bodies emerge at the end of winter or in early spring.

### Beyond the definition: practical considerations

As indicated in the section above, a major issue with the definition and use of the term dormancy in fungal biology is the historical link with plant science and the concept of dormancy as being solely the delay in germination under favorable conditions. Accordingly, dormancy in fungal literature is often associated with the capability of a propagule (i.e., spore, chlamydospore, and sclerotia) to germinate. In its seminal paper on fungal dormancy, Cochrane (6) describes the concept of constitutive dormancy, a condition in which development is delayed due to an innate property of the dormant cellular stage. This concept is illustrated by the germination of the ascospores of *Neurospora*, which cannot germinate until its internal stores of the sugar trehalose have been consumed. This is triggered upon activation by heat, in which the required enzyme enters in contact with its substrate. A second example provided by this author is the degradation of self-inhibitors in asexual spores, which avoid germination when multiple spores are confined in the same space. Both examples respond to a pragmatic approach to the definition of dormancy by focusing on what could be measured and tested to assess the level of dormancy. However, exogenous dormancy as an additional concept for dormancy in fungi (7) is a closer fit to the overall definition proposed by McDonald et al. (2), which also considers the notion of retaining viability under unfavorable environmental conditions.

Finding common characteristics, such as morphological, chemical, or physical features, to different types of dormant structures could be another way to study them, which may lead to reliable methods. However, due to the high variation in the type of dormant cells and their associated morphophysiological features, finding universal morphological or chemical markers is unlikely. As a valuable comparison, a study assessing chemical and morphological characteristics of different types of bacterial resistant cells showed that the only common morphological characteristic to diverse structures was the enlargement of the cell envelope, while no common chemical markers were found using Raman microspectroscopy (64). The larger diversity of potential dormant cells highlighted earlier in the text suggests an even more challenging problem in the case of fungal dormancy. Therefore, future advancements in the understanding of fungal dormancy will go hand in hand with methodological progress allowing investigation of the different stages of dormancy. For this, presenting the state of the art of methods applied to bacterial dormant structures generates a useful comparison. Moreover, to facilitate their presentation, the methods are organized according to the different phases required for the generation of dormant structures.

#### Assessing spore morphology

In filamentous fungi, identifying spores is straightforward when spores are formed on a conidiophore or in a sporangium, which show a clear differentiation from the vegetative hyphae. The same applies to sexual spores (ascospores, basidiospores, and zygospores), which are all associated with very specific structures. The latter are so specific that the microscopic morphological characteristics of these spores and their associated structures are used for taxonomic classification (65). However, other fungal dormant structures such as chlamydospores or spores formed by yeasts are much more difficult to distinguish. This is also the case for any spore that is not associated with a multicellular structure that gives rise to the spore (e.g. conidiophore, basidium, and ascus). Likewise, sclerotia (including micro- and pseudosclerotia), depending on their size and morphology and the environment where they form, may be difficult to identify and differentiate from the environmental matrix.

Despite the challenges of identifying dormant cells or structures using microscopy, certain morphological markers are characteristic of specific cell types and can be targeted using appropriate methods. A well-known example is the detection of bacterial endospores via phase-contrast microscopy, where endospores appear as bright, refractile bodies due to their unique optical properties (66, 67). Likewise, two experimentally useful criteria of myxospore induction are the conversion of vegetative rods to optically refractile short rods or ovoids and the development of resistance to sonic lysis (68). Similarly, some fungal spores, such as those of *S. cerevisiae*, also exhibit light refractility and can be visualized using the same techniques (69). However, and most importantly, differentiating fungal vegetative cells from dormant structures is doable in pure culture of well-characterized organisms but is much more difficult in samples consisting of mixed cultures or within an environmental matrix.

#### Metabolic status

Distinguishing between active and dormant states among viable cells introduces an additional layer of complexity to the already problematic issue of defining whether individual cells are alive or dead (70). Staining techniques, such as live/dead assays based on membrane integrity, have traditionally been used to assess microbial cell viability in various sample types, including those containing dormant cells. These methods are often combined with flow cytometry or epifluorescence microscopy. However, their effectiveness is limited when applied to resistant cells, such as spores, due to the inherent robustness of their cell envelope, which can prevent stain penetration. Considering the connection between dormancy and the need to protect the cells from external stressors, studying dormancy is intrinsically challenging due to the increased resistance of dormant cells. Accordingly, many common viability stains fail to label spores effectively (70). Moreover, while these methods can distinguish between live and dead cells, they cannot reliably differentiate between active and dormant states. As such, although useful as visual tools, staining methods alone are insufficient for studying the metabolic status of dormant cells (71). Photoreactive DNA dyes, such as propidium monoazide (PMA), offer an alternative approach. PMA is one of the most frequently applied non-membrane-permeating dyes, since PMA dye penetrates only cells with damaged membranes and not cells with intact membranes. Upon photoactivation, the dye intercalates with DNA and inhibits downstream amplification from DNA of damaged cells (70). Combined with quantitative PCR (qPCR), this method has been used to quantify the viability of fungal spores in environmental samples. Two studies exemplified the use of this method to quantify the viability of conidia from diverse fungal pathogens in different environmental matrixes such as air, water (72), or soil (73).

Other approaches involve measuring metabolic activity, such as ATP levels or respiration rates, to assess the overall activity within a sample and determine whether microorganisms are metabolically active. However, when applied to environmental or mixed-community samples, these methods provide only a bulk estimate of activity and cannot distinguish between specific taxa or cell types. To gain more taxonomic resolution, RNA-based approaches, including RNA extraction and sequencing, can be employed to identify which microbial genera or species are transcriptionally active (70). The usefulness of this approach was presented early in the case of the study of developing conidia (32). Similar studies could reveal more specific methods to detect the metabolic status of dormant fungal cells. Stable isotope probing (SIP) offers a complementary strategy by enabling the labeling of newly synthesized DNA or RNA. This technique can detect low levels of metabolic activity and provide insights into the active fraction of microbial communities, even under conditions where traditional methods may fail (70).

#### Resuscitation

Assessing the environmental factors that trigger the exit from dormancy, as well as the subsequent survival of microbial cells, is still often achieved using traditional culture-dependent approaches. These include culture-based germination and viability assays, which are applicable to both bacterial and fungal dormant structures. Despite the rise of molecular techniques, cultivation-based assays remain essential for determining the viability of dormant cells (70). While such methods typically assess populations of cells simultaneously, recent advances, such as microfluidic devices coupled with microscopy, allow for the monitoring of individual cell behavior (74). Additionally, the transition from dormancy to active growth in fungi can involve molecular chaperones, such as heat shock proteins (HSPs), with the example of Hsp42, which plays a role in the germination of *S. cerevisiae* ascospores (69, 75). Investigating such molecular markers can provide valuable insights into the mechanisms underlying dormancy and resuscitation and may offer tools for detecting dormant cells in environmental samples.

#### Protection from decay

A common hallmark of the formation of dormant structures in bacteria and fungi is the incorporation of additional protective layers and the incorporation of specific metabolites to modify the fluidity of the cytoplasm and to protect the genetic material of the cell to ensure protection from decay (Table 1). While for some groups the chemical composition of the dormant cell represents a unique chemical signature, such as in the case of the presence of dipicolinic acid in endospores (64), other compounds such as compatible solutes, including polyols (glycerol, mannitol, arabitol, and erythritol) or the disaccharide trehalose, and pigments are found in different groups (33, 64). Incorporation of trehalose has been correlated with higher temperature resistance in fungi (35). A more specific compound that is found in the walls of zygospores is sporopollenin, a highly resilient and chemically stable biopolymer that provides protection against degradation and stress (76). Chitin, beta-glucans, and melanin, which are common components of the fungal cell wall, can also be found in fungal spores. However, the distribution and chemical conformation of these components can vary between the vegetative mycelium and spores. Fungal chitin is considered a common and typical component of the fungal cell wall of mycelia and spores, but it is not found in all life stages and all fungi and may be entirely absent. For instance, the cell wall of spores of *Trichoderma viride* shares with the mycelial cell wall polymers such as beta-(1,3),beta-(1,6) glucans, and mannoproteins, but chitin, which is always present in the mycelium, is replaced by chitosan and melanin in the spore wall (77, 78). Finally, it is interesting to note that sclerotia may contain chemically diverse compounds with biological activities, such as the case of the high alkaloid content in *Claviceps purpurea* (79). These compounds likely have a role in the resistance of these structures to fungivory or degradation by other microbes (80). Similarly, sclerotia of *Aspergillus flavus* have been shown to sequester secondary metabolites acting as a chemical defense system against fungivorous predators, allowing these structures to become a primary source for inoculation of this phytopathogenic fungus (80). As a result, some of these compounds, owing to more research on their presence in different fungi, may become interesting targets to assess a potential for dormancy.

**TABLE 1:** Main features of dormant structures in fungi.^*a*^

Structure	Group	Model example	Morphogenesis	Distinct morphological features	Key chemical components	Compatible solutes/special compounds	Resistance factors	Survival advantage(s)
Ascospores	Ascomycota		Nuclear fusion in a dikaryotic cell compartment, meiosis and mitosis within an ascus	Enclosed in asci	Chitin, melanin, trehalose, mannite	Trehalose, mannitol	UV, heat, desiccation	Sexual reproduction, genetic diversity
Basidiospores	Basidiomycota		Nuclear fusion in a dikaryotic cell, the future basidium, followed by meiosis and nuclei migration in basidiospores	Produced on basidia	Chitin, melanin, lipid bodies	Trehalose, glycerol	UV, desiccation	Dispersal, sexual reproduction
Zygospores	Zygomycota s.l.		Fusion of modified hyphal apices called gametangia	Thick-walled resting spore	Thick chitin wall, carotenoid-derived sporopollenin	Trehalose, lipids	Cold, desiccation	Dormancy, sexual reproduction
Conidia	Dikarya	*Aspergillus nidulans* and *Neurospora crassa*	Mitotic divisions and periodic budding or fission cycles of a conidiogenic cell	Asexual spores, often airborne	Chitin, melanin, lipid droplets	Trehalose, glycerol, proline	Heat, UV, desiccation	Rapid dispersal, colonization
AMF spores (glomerospores)	Glomeromycotina	*Rhizophagus irregularis*	Terminally or intercalary, singularly or in clusters, on sporogenic hyphae. Nuclei migration in sporangia followed by mitosis to generate a multinucleated spore.	Phenotypically plastic, multi-layered envelope	Microfibrils (crystalline chitin, beta-glucans) embedded in an amorphous matrix., melanin.	Trehalose, lipids	Desiccation, temperature extreme, heavy metals, salinity	Dormancy until finding a suitable host (obligate biotrophs)
Chlamydospores	Fungi	*Candida albicans*	Cytoplasmic condensation within a specialized cell	Thick-walled resting spore	Thick chitin wall, lipid inclusions	Trehalose, glycerol	Nutrient deprivation, desiccation	Dormancy, survival in host or environment
Sclerotia (tissue)	Dikarya	*Sclerotinia* spp.	Aggregation, interweaving, and branching of hyphae	Hardened mass of mycelium	Dense mycelial matrix, melanin, storage lipids	Trehalose, mannitol	Cold, desiccation	Long-term survival, regrowth
Endospores	Firmicutes	*Bacillus subtilis*	Asymmetrical cell division resulting in the formation of a mother cell and the endospore	Multi-layered: exosporium, spore coat, cortex, core	Dipicolinic acid (DPA), calcium ions, SASPs, peptidoglycan, exosporium proteins/lipids	DPA-Ca²⁺ chelate, pyridine-2,6-dicarboxylic acid	Heat, radiation, desiccation, chemicals	Long-term dormancy, extreme environmental survival
Exospores	Actinobacteria	*Streptomyces* spp.	Synchronic division of specialized aerial hyphae	Formed externally on aerial hyphae	Thick peptidoglycan wall, lipid-rich membrane, trehalose	Trehalose, ectoine	Desiccation, UV, moderate heat	Dispersal, dormancy, survival in soil environments
Myxospores	Myxococcales	*Myxococcus* spp.	Differentiation of cells within a fruiting body	Formed in fruiting bodies	Thickened envelope, reduced water content	Trehalose, glycine betaine	Starvation, desiccation	Social survival strategy, dispersal
Cysts	Azotobacteracea	*Azotobacter* spp.	Enlargement of the cell wall	Thick outer wall	Lipid-rich outer layer, polysaccharides	Trehalose, proline, glutamate	UV, desiccation, chemicals	Dormancy, survival in soil
Akinetes	Nostocales and Stigonematales	*Nostoc* spp.	Development of a thick, protective multilayered envelope	Enlarged vegetative cells	Thick cell wall, storage granules	Sucrose, trehalose, glucosylglycerol, glycine betaine	Cold, nutrient limitation, desiccation	Seasonal survival, regrowth in favorable conditions

^*a*^ Bacterial spores are shown for comparative purposes.

As mentioned above, a very well-established example of the use of chemical markers to detect bacterial dormant cells is the detection of calcium dipicolinic acid (CaDPA), a molecule that contributes to the heat resistance of bacterial endospores. The presence of CaDPA in diverse samples can be detected using techniques such as Raman microspectroscopy (64) or high-performance liquid chromatography (HPLC), making it a reliable biomarker for identifying bacterial endospores (81). Currently, comprehensive studies aiming at identifying similar chemical markers in resistant fungal cells are lacking. Nonetheless, research on specific fungal groups, such as arbuscular mycorrhizal fungi (AMF) or model species like *S. cerevisiae*, has revealed distinct biochemical features. For example, certain fungal spores accumulate high concentrations of protective molecules such as trehalose (75, 82) and mannitol, while the ascospores of some yeasts possess an acidic and viscous cytoplasm (69). Those molecules could be the basis for the detection and quantification of fungal spores. However, as they are not specific (Table 1), this could result in methods that will only provide a very unspecific proxy to dormancy in fungi.

Reliable detection techniques for some fungal dormant structures, such as sclerotia, are still lacking. Since melanin is a prevalent compound of hyphae making up sclerotia, this compound could be a good proxy to sclerotia. However, melanin is present in other types of fungal cells, and melanin itself is a challenging molecule to detect and quantify in environmental samples (83).

#### Other methodological considerations

Given the intrinsic need for protecting the cell content during dormancy, specific methodological considerations are needed for molecular studies of dormant cells, as physically accessing their DNA can be challenging. Accordingly, targeted DNA extraction methods have been developed to extract DNA from bacterial endospores (84), as well as from different types of fungal spores (85). Such methods rely both on the physical and chemical resistance to lysis of different cell types. Some resistant fungal structures, such as spores, melanized cells, or sclerotia, were shown to be as lysis-resistant as bacterial spores, and thus the lysis-resistant characteristic of these types of cells can be used to assess both bacterial (86) and fungal (85) seed banks. These methods enable the detection of lysis-resistant cells within environmental samples, providing both diversity and quantitative insights when used in combination.

Another approach for identifying and quantifying resistant bacterial cells involves targeting dormancy-specific genes. This is possible when there is a deep understanding of the process, as in the case of endospore formation. Highly conserved sporulation-specific genes, such as *spo0A*, are prime candidates for the development of specific detection methods (87, 88). Although the presence of such genes does not distinguish between viable and non-viable cells, it allows for the assessment of the potential presence of endospores in communities and enables the detection of their producing taxa (i.e., Bacillota). A similar approach could be applied targeting key master regulators for different phases of the cell cycle in fungi.

For instance, three genes *brlA*, *abaA*, and *wetA*, have been proposed to correspond to the central regulatory pathway that coordinates conidiation in *A. nidulans*. The product of the first of these genes, BrlA, is a transcriptional factor required for the transition from polarized apical growth of the conidiophore stalk. AbaA, whose gene is activated by BrlA in the middle stages of conidiophore development, is an essential component for the proper differentiation and function of phialides. Finally, *wetA* is induced by AbaA in the late phase of conidiation, and its product plays an important role in the synthesis of cell wall layers that make conidia mature and impermeable (89). However, a search for homologs in the protein database UniProt yielded no homologs for either of the three proteins at 90% identity, but multiple hits at 50% identity (e.g., 185 entries for AbaA). Another set of genes involved in the regulation of conidiogenesis are the *velvet* regulators, which are recognized as coordinating growth, differentiation, and secondary metabolism (89, 90). The search for homologs with 50% identity yielded multiple hits. Another set of genes that could be of interest are those jointly regulating secondary metabolism and sporulation. Among the potential candidates for common regulatory pathways are the G signaling proteins encoded by genes such as *fadA* or *ganB*, to name but two, which commonly regulate fungal development (91). Another set of genes that have been shown to be relevant for sporulation in *Aspergillus* and *Neurospora* are the *con* genes, some of which have been shown to be expressed in the three sporulation pathways of *N. crassa* (i.e., micro- and macro-conidiation, sexual reproduction) (92). Therefore, designing gene models and associated PCR primers could be considered in the future to detect those genes in environmental samples and develop culture-independent methods to assess some types of fungal sporulation (here mostly conidiation). Likewise, with the advances in sequencing technologies, identification of the molecular mechanisms involved in the control of sclerotia formation may highlight gene clusters that could be used as markers (93).

### Environmental relevance of fungal dormancy

While studying dormancy under laboratory conditions is essential to understand its mechanisms, developing approaches to investigate dormant cells in environmental samples under conditions conducive to dormancy (i.e., fluctuating environments) is equally important. This raises the question of which ecosystems could serve as interesting *in situ* laboratories for studying dormancy.

The importance of the fluctuation of environmental conditions to induce dormancy over extreme conditions can be highlighted by the role of dormancy in community recovery in soils, in which only a very small fraction of the community is considered to be active (below 2%) (94). For instance, mesocosm experiments have shown that, together with dispersal, dormancy plays an important role in the resilience of microbial communities subjected to thermal variations (95). Indeed, soils represent environments in which dormancy is a common survival strategy due to fluctuating environmental conditions and the high heterogeneity of the environmental matrix (96). The ecological and evolutionary success of fungi in soils is due to their unique lifestyle and physiology. Filamentous fungi can explore solid substrates and are thus uniquely suited for life in soil. Their hyphae function as bidirectionally transport systems across soil microenvironments (97).

Soil may be considered one of the most representative environments for fungi (98); however, one should not forget the likely marine origin of fungi. Ancestral fungi must have been primarily aquatic (99), and therefore, finding environmental analogs to those early aquatic environments could be relevant to investigate the evolutionary history of dormancy in fungi. Therefore, we would like to make the case for studying fungal dormancy in extreme ecosystems accessible by man-made infrastructures, such as deep geothermal reservoirs (100, 101). Poly-extreme environments, where multiple stressors coexist, are especially intriguing because cells must withstand several limiting factors simultaneously, potentially resulting in exceptional survival strategies. Examples of poly-extreme environments include high-altitude salterns, deep-sea anoxic basins, and geothermal fluids of typically high temperatures, high pressures, and high salinities. However, poly-extreme environments are not *per se* conducive to dormancy unless conditions are highly fluctuating. This is typically the case of environments linked to industrial processes, where abiotic factors vary dynamically (e.g. temperature in the agrifood industry) and where high resistance of fungi has been reported (33, 35).

Among poly-extreme environments, our research has pointed to subsurface geothermal reservoirs as interesting systems in which to study both fungal and bacterial dormancy. During our research, we have isolated several bacterial and fungal species from deep geothermal waters, all of which have in common the production of dormant cells (102–105). Deep geothermal reservoirs below the surface (up to several km) combine multiple extremes: high temperatures, variable pH and salinity, and anoxic conditions, making them representative of polyextreme environments (101). More importantly, geothermal fluids are increasingly exploited for heat and electricity provision, or to act as heat-storage systems. These activities involve purposeful manipulation of environmental conditions creating highly fluctuating conditions. Therefore, understanding the response of dormant cells to changes in environmental conditions could help, for instance, to prevent the revival of undesirable microorganisms. It is well known that the activation of H₂S-producing microbes during fluid exploitation can lead to severe corrosion problems in geothermal facilities (106). This can inform strategies for the sustainable use of geothermal energy (101).

**Fig 3:**
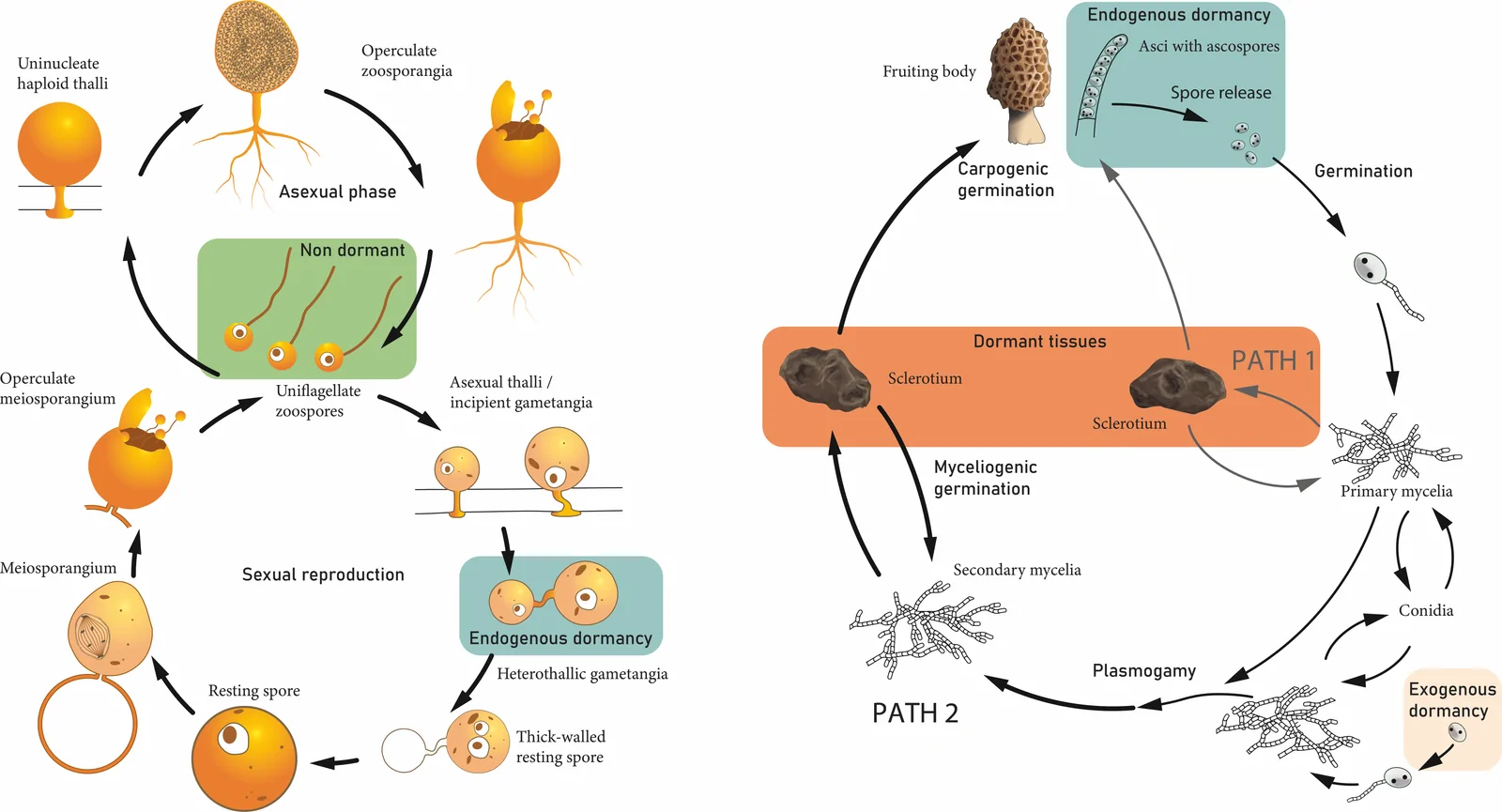
Comparison of the life cycle of an aquatic fungus (based on *Zygorhizidium planktonicum*) and a terrestrial one (based on *Aspergillus nidulans*) highlighting the differences in the dormant cells and structures between these two habitats. While in both groups the presence of spores with an endogenous (i.e., breaking of dormancy by overcoming cellular barriers) dormant phenotype exists, the asexual spores in each life cycle contrast regarding dormancy. Zoospores are non-dormant motile cells that lack a cell wall and swim with a motile cilium. In contrast, conidia (in known life cycles of typically terrestrial fungi) are known to exhibit exogenous dormancy (i.e., breaking of dormancy when an unfavorable environmental factor ceases). Likewise, the emergence of dormant tissues such as sclerotia is known for terrestrial fungi but not in aquatic ones. The depiction of the life cycle in *Z. planktonicum* is based on reference (110).

Geothermal environments are also interesting from an evolutionary perspective. For instance, the last bacterial common ancestor (LBCA) is thought to have been a thermophile thriving under anaerobic conditions (107, 108), positioning deep geothermal waters as potential analogs to early Earth habitats. Sporulation, which is considered an ancient survival trait, likely enabled life to persist during these primordial conditions (108). Therefore, studying microorganisms in deep geothermal waters offers an opportunity to explore the origins and evolution of dormancy strategies. Such research can help identify common determinants of dormancy. Importantly, these environments also provide an exceptional opportunity to investigate dormancy in fungi, a largely overlooked aspect in extreme microbiology (109). Inferring the potential lifestyle of the last common fungal ancestor (LCFA) is challenging. The sister group to fungi are amoeboid Nucleariida protozoans that are common in marine environments. Now, most of the known fungal diversity is found in terrestrial ecosystems, to which fungi rapidly adapted upon the inferred loss of the flagellum providing motility in aquatic environments (99). In extant clades of aquatic fungi such as chytrids, zoospores are not dormant (38) and can be considered as dispersal units that have been compensated and are functionally equivalent to hyphal filamentous growth in terrestrial fungi (99). As a result, one could wonder if the evolution of new types of dormant cells and structures (e.g., sclerotia or chlamydospores), from the ancestral resting sporangia, was not a crucial feature that allowed fungi to colonize terrestrial environments (Fig. 3). This type of hypothesis could be tested in the future by increasing our knowledge of fungal diversity in extreme aquatic ecosystems and, for instance, by reconstructing fungal genomes from deep geothermal environments.

### The many faces of microbial dormancy

A major drawback of the large diversity of dormant cells and tissues in bacteria and fungi is the difficulty to find an all-encompassing dormancy definition. The confluence of morphophysiological traits required to overcome adverse conditions can result in the production of highly specialized dormant cells (11). This is the case for recognized examples of dormant structures in bacteria such as endospores (111, 112), exospores (22, 113), myxospores (68, 114), akinetes (115), or cysts (42, 116), but also sexual and asexual fungal spores, chlamydospores, resting sporangia, and sclerotia. These specialized cells and tissues do not share a common evolutionary origin and are generally specific to defined phylogenetic groups. The diversity of these structures and the fact that they do not necessarily share a common chemical or morphological signature (64) attest to the difficulties of determining the existence of a specialized dormant cell in a specific microbial group but suggest that the production of specialized cells could be a unifying element of dormancy across diverse microorganisms. As spores are the most easily recognizable and tractable dormant cells produced by microorganisms, following different sporulation processes in diverse microorganisms, from archaea to oomycetes, could reveal some of the features of a common definition of dormancy that encompasses diverse cells.

The complexity and multitude of dormant cells produced during diverse microbial life cycles are often understated. This was illustrated here by the formation of diverse dormant cells and tissues in fungi. However, fungi are by far not the only microbial eukaryotes in which dormant cells are relevant in their life cycles. For instance, many oomycetes (filamentous stramenopile or pseudofungi) produce oospores that, after a period of development, require a period of obligate dormancy to germinate (117). Also, pathogenic oomycetes such as *Phytophthora cinnamomi* are known to produce chlamydospores that are relevant as survival dormant structures in soils (118). There are other prominent examples that can be considered akin in complexity, such as the formation of fruiting bodies and myxospores inside a starving *Myxococcus* bacterial colony (114). The link between life cycle and dormancy appears not to be exclusive to fungi, oomycetes, or bacteria. A group of archaea from the *Halobacteriaceae* was shown to display a life cycle that resembles that of the actinobacterial genus *Streptomyces*, thus undergoing cellular differentiation of hyphae and spores. A newly described representative species of this group, *Actinarchaeum halophilum*, which was isolated from a salt marsh, provides evidence of common evolutionary and genetic components to cellular differentiation programs across different domains of life (119). Furthermore, realizing that spores are but one of the dormant cell types produced by microorganisms in their life cycles also opens the possibility to define dormancy in a broader sense. For example, experiments performed with *Sulfolobus* spindle-shaped virus (SSV9) and its native archaeal host, *Sulfolobus islandicus*, have demonstrated that exposure to active and inactive particles triggers a significant growth delay that results in a viable, non-growing dormant state (120). The relationship between viruses and dormant cells is not exclusive to archaea, and it has been long investigated in bacteria as well (121).

As a survival strategy, levels of dormancy, activity, and state-switching vary across individual cells and can be directly connected to the life cycle stages of a given species. Importantly, how should one consider cells that have very low activity rates and that are neither considered active nor dormant (122)? For instance, colony morphology switching, which refers to the phenotypic plasticity of growth forms of fungi, is typically observed in isolated populations and allows for a better ecological fitting to specific habitats (123). While this is initially a transient state, it may lead to the establishment of “persistent ecotypes” over evolutionary timescales in environments where survival strategies are central. Black yeasts such as *Aureobasidium pullulans* mitigate the impact of environmental stressors by rapidly switching from small, colorless yeast cells to colonies in which heavily melanized, thick-walled cells reproduce by isodiametrical division, also called meristematic growth. This results in a colony morphology that protects the organism against environmental stresses and supports survival (124). Indeed, meristematic growth allows energy preservation and metabolic activity while minimizing surface area, which leads to better survival under extreme environmental conditions like desiccation (125). Moreover, heavily melanized, thick-walled cells are more resistant to environmental stressors compared to non-melanized cells (126, 127). Dothidealean black yeast-like fungi are another good example of a group in which meristematic growth allows survival. In this case, cell fragments constantly detach from the colony and colonize new habitats. The fixation of this growth mode has been associated with environmental stress in some species and could be considered an adaptive strategy for survival.

Is this type of growth plasticity resulting in a dormant structure? No, persistent ecotypes in fungi, including colony morphology switching or melanized meristematic growth, do not result in a dormant structure. They correspond to active, stress-adapted phenotypic states rather than representing a dormancy strategy. For instance, the fact that meristematic growth allows the colony to continue growing would be against the idea of considering this survival strategy a form of dormancy and would rather be an alternative mechanism to respond to unfavorable environmental conditions, as in the case of dimorphism (128). Nonetheless, this growth type shares some functional similarities with dormancy strategies, such as bacterial persisters. As in the case of bacterial persisters, persister ecotypes in fungi enhance survival under stress conditions. However, while persister bacterial cells are non-dividing or growth-arrested, persistent fungal ecotypes continue dividing, even if very slowly (129). They emerge stochastically or, for instance, in response to antibiotics, which trigger the arrest of reproductive growth and the expression of genes for enzymes in biosynthetic pathways, yielding resistance to antagonistic compounds and complicating antibiotic treatment (130, 131). By systematically comparing known structures and survival strategies, we could start developing approaches to identify new structures and even to detect dormant cells in taxa where such structures have not yet been described (64).

### Conclusion and outlook

Microbial dormancy is a fundamental survival strategy with profound implications for ecosystem resilience, microbial diversity, and biogeochemical cycling. At the ecosystem level, dormancy contributes to the maintenance of microbial diversity and facilitates recovery following environmental disturbances (1, 3). Traits associated with dormancy, such as the ability to form resistant structures, have been identified as key factors in the success of microbial colonizers in experimental systems (132). However, the ecological and functional significance of fungal dormant forms outside of fungal pathogens requires further investigation. Fungal dormancy is particularly relevant in applied contexts. In food systems, dormant fungal cells pose persistent challenges for food spoilage prevention and food safety (33, 133). In water distribution networks, fungal biofilms can lead to infrastructure degradation and potential health risks (134). Even in highly controlled environments, such as spacecraft wastewater systems, the focus has largely been on bacterial dormancy (135), overlooking the potential resilience and persistence of fungal cells. This is particularly surprising given the famous case of the “*kerosene fungus*,” which led to the immobilization of aircraft in the 50s due to fuel degradation, filter clogging, and induced corrosion in pipes and tanks (136). Moreover, fungal dormancy plays a critical role in agriculture and human health. Sclerotia of plant pathogenic fungi can persist in soils for extended periods, complicating disease management but also offering a reservoir of bioactive compounds with potential applications in medicine and agriculture (52). In clinical settings, latent fungal infections, such as those caused by *Cryptococcus neoformans*, highlight the ability of fungal spores to remain dormant within human hosts for years (137, 138). The reactivation of those dormant spores in immunocompromised individuals has fatal consequences. Finally, the occurrence of dormant microorganisms in deep geothermal reservoirs and the exploitation of these reservoirs for energy production bears risks because of the undesired growth upon change of the extreme conditions, but they also hold relevant information for the understanding of the early history of life evolution. Given the implications, it is imperative that future research expands beyond bacterial models to include the rich and varied strategies of fungal dormancy, and eventually of dormancy in other microbial groups. This will require the development of new tools and conceptual frameworks capable of capturing the complexity of dormancy across microbial domains.

## Supplementary Material

Reviewer comments

## SUPPLEMENTAL MATERIAL

OPEN PEER REVIEW reviewer-comments.pdf An accounting of the reviewer comments and feedback.

ASM does not own the copyrights to Supplemental Material that may be linked to, or accessed through, an article. The authors have granted ASM a non-exclusive, world-wide license to publish the Supplemental Material files. Please contact the corresponding author directly for reuse.
